# β-Xenophyllite-type Na_4_Li_0.62_Co_5.67_Al_0.71_(AsO_4_)_6_

**DOI:** 10.1107/S1600536813025233

**Published:** 2013-09-21

**Authors:** Riadh Marzouki, Wafa Frigui, Abderrahmen Guesmi, Mohamed Faouzi Zid, Ahmed Driss

**Affiliations:** a Laboratoire de Matériaux et Cristallochimie, Faculté des Sciences de Tunis, Université de Tunis ElManar, 2092 Manar II Tunis, Tunisia

## Abstract

The title compound, tetrasodium lithium cobalt aluminium hexa­(orthoarsenate), was synthesized by a solid state reaction route. In the crystal structure, Co^2+^ ions are partially substituted by Al^3+^ in an octa­hedral environment [*M*1 with site symmetry 2/*m*; occupancy ratio Co:Al = 0.286 (10):0.714 (10)]. The charge compensation is ensured by Li^+^ cations sharing a tetra­hedral site with Co^2+^ ions [*M*2 with site symmetry 2; occupancy ratio Co:Li = 0.690 (5):0.310 (5)]. The anionic unit is formed by two octa­hedra and three tetra­hedra linked only by corners. The Co*M*1*M*2As_2_O_19_ units associate to an open three-dimensional framework containing tunnels propagating along the *a-*axis direction. One Na^+^ cation is located in the periphery of the tunnels while the other two are situated in the centres: all Na^+^ cations exhibit half-occupancy. The structure of the studied material is compared with those of various related minerals reported in the literature.

## Related literature

For applications of these and related phases, see: Aurivillius *et al.* (1964 ▶); Nagpure *et al.* (2010 ▶); Prabaharan *et al.* (1997 ▶). For details of structurally related compounds, see: Alvarez-Vega *et al.* (2006 ▶); Keller *et al.* (1981 ▶); Frigui *et al.* (2012 ▶); Goodenough *et al.* (1976 ▶); Marzouki *et al.* (2012 ▶); Ben Smida *et al.* (2013 ▶); Guesmi & Driss (2012 ▶); Moring & Kostiner (1986 ▶); Kobashi *et al.* (1998 ▶); Ben Smail *et al.* (1999 ▶); Burke *et al.* (2006 ▶); Redhammer *et al.* (2005 ▶); Hatert *et al.* (2005 ▶); Moore & Molin-Case (1974 ▶). For the bond-valence method, see: Brown & Altermatt, (1985 ▶).

## Experimental

### 

#### Crystal data

Na_4_Li_0.62_Co_5.67_Al_0.71_(AsO_4_)_6_
*M* *_r_* = 1283.06
Monoclinic, 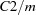

*a* = 10.7444 (9) Å
*b* = 14.847 (2) Å
*c* = 6.7223 (8) Å
β = 105.51 (2)°
*V* = 1033.3 (2) Å^3^
*Z* = 2
Mo *K*α radiation
μ = 14.22 mm^−1^
*T* = 298 K
0.26 × 0.24 × 0.22 mm

#### Data collection

Enraf–Nonius CAD-4 diffractometer
Absorption correction: ψ scan (North *et al.*, 1968 ▶) *T*_min_ = 0.033, *T*_max_ = 0.042
1615 measured reflections
1174 independent reflections
1048 reflections with *I* > 2σ(*I*)
*R*_int_ = 0.027
2 standard reflections every 120 min intensity decay: 1.4%

#### Refinement

*R*[*F* ^2^ > 2σ(*F* ^2^)] = 0.026
*wR*(*F* ^2^) = 0.068
*S* = 1.13
1174 reflections
117 parameters
2 restraints
Δρ_max_ = 0.87 e Å^−3^
Δρ_min_ = −0.87 e Å^−3^

### 

Data collection: *CAD-4 EXPRESS* (Duisenberg, 1992 ▶; Macíček & Yordanov, 1992 ▶); cell refinement: *CAD-4 EXPRESS*; data reduction: *XCAD4* (Harms & Wocadlo, 1995 ▶); program(s) used to solve structure: *SHELXS97* (Sheldrick, 2008 ▶); program(s) used to refine structure: *SHELXL97* (Sheldrick, 2008 ▶); molecular graphics: *DIAMOND* (Brandenburg, 1998 ▶); software used to prepare material for publication: *WinGX* (Farrugia, 2012 ▶).

## Supplementary Material

Crystal structure: contains datablock(s) I. DOI: 10.1107/S1600536813025233/vn2076sup1.cif

Structure factors: contains datablock(s) I. DOI: 10.1107/S1600536813025233/vn2076Isup2.hkl

Additional supplementary materials:  crystallographic information; 3D view; checkCIF report

## Figures and Tables

**Fig. 1.**
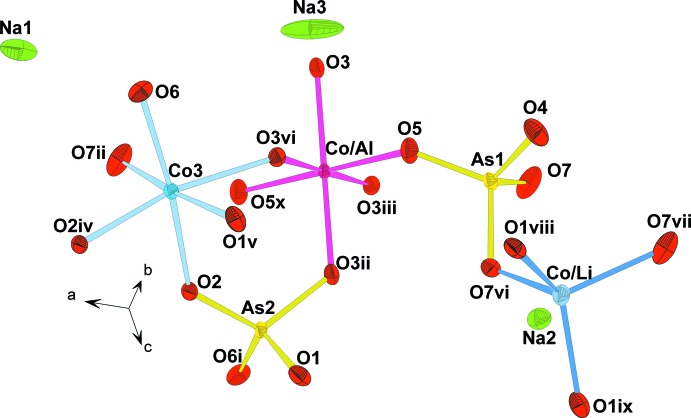
L'unité asymétrique dans Na4Li0,62Co5,67Al0,71(AsO4)6. Les éllipsoïdes ont été définis avec 50% de probabilité. Codes de symétrie: (i) x, y, z + 1; (ii) x, y + 1, z; (iii) x, -y, z; (iv) -x + 3/2, -y + 3/2, -z + 1; (v) -x + 1, y, -z + 1; (vi) -x + 1, y + 1, -z + 1; (vii) x - 1, y + 1, z; (viii) x - 1/2, -y + 3/2, z; (ix) -x + 1/2, -y + 3/2, -z + 1; (x) -x + 1, -y, -z + 1.

**Fig. 2.**
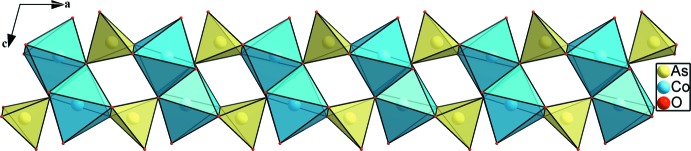
Vue selon b, montrant la jonction des dimères Co2O10 avec les tétraèdres As2O4.

**Fig. 3.**
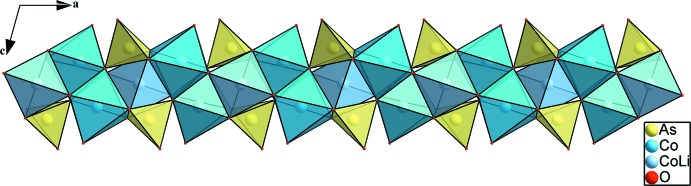
Projection d'un ruban dans le plan ac.

**Fig. 4.**
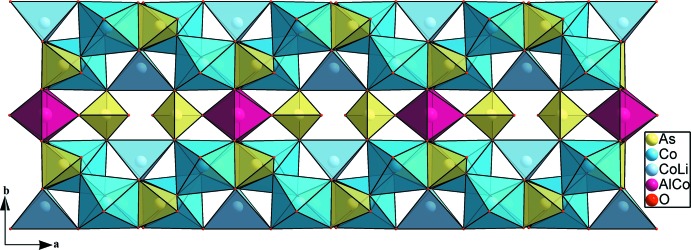
Vue selon c, montrant la cohésion entre les rubans.

**Fig. 5.**
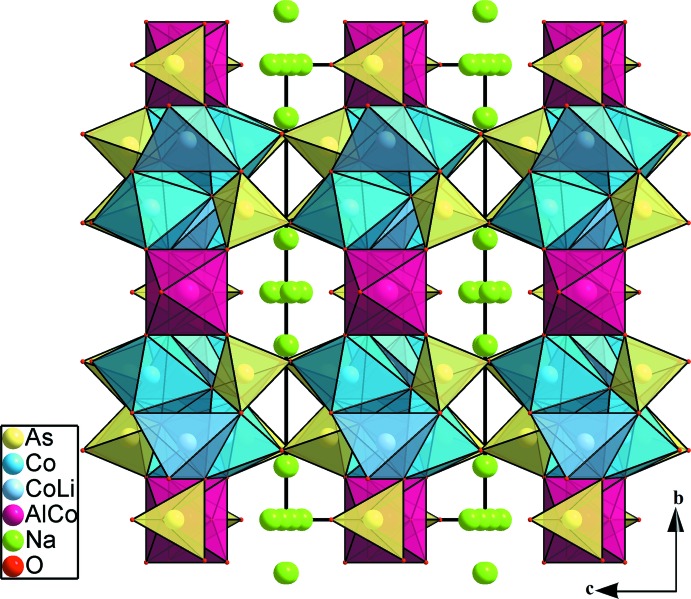
Vue selon c, montrant la jonction entre les couches.

**Fig. 6.**
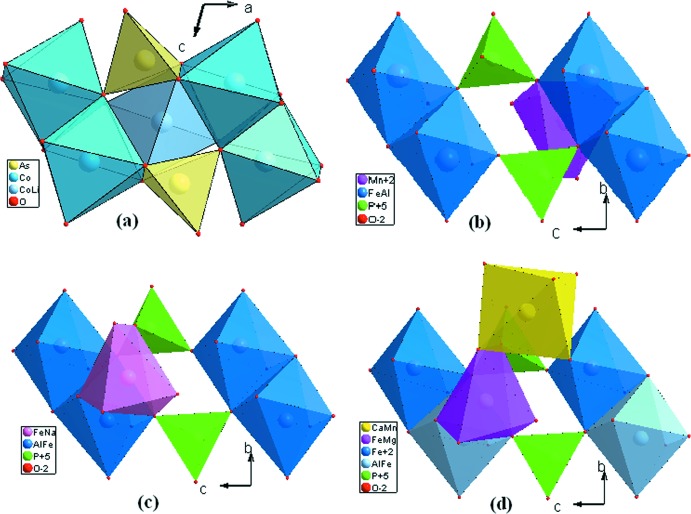
Projection montrant la jonction des dimères dans: (a): le matériau Na4Co4(Co0.688Li0.309)2(Co0.285Al0.711)(AsO4)6, (b): l'alluaudite (Na0.84Ca0.32)Mn(Fe1.74Al0.26)(PO4)3, (c): le rosemaryite (Mn0.366Na0.325)(Fe0.911Na0.088)(Al0.708Fe0.296)(Fe0.791Al0,215)(PO4)3 et (d): le wyllieite Na4.6(CaMn)Fe4(Fe0.75Mg0.25)4(Fe0.25Al0.75)4(PO4)12.

## supplementary crystallographic information

### 1. Comment

Les phosphates et les arséniates de métaux de transition ont un champ prometteur pour diverses applications: ferroélectriques, magnétiques (Aurivillius *et al.*, 1964; Nagpure *et al.*, 2010). Néanmoins, l'introduction d'ions monovalents dans ces oxydes peut conduire à des matériaux ayant des propriétés intéressantes, en particulier de conduction ionique (Prabaharan *et al.*, 1997).

Selon cette approche, les phosphates et arséniates mixtes de métaux de transition et de cations alcalins sont étudiés. Ces matériaux présentent une richesse structurale remarquable: type olivine (Alvarez-Vega *et al.*, 2006), type alluaudite (Keller *et al.*, 1981), structure wyllieite (Frigui *et al.*, 2012). En relation avec leurs structures, ces matériaux présentent de nombreuses propriétés physico-chimiques: conduction ionique, échange d'ions (Goodenough *et al.*, 1976).

Dans ce contexte, nous avons tenté d'explorer les systèmes *A*_2_O–CoO–Al_2_O_3_–*X*_2_O_5_ (*A* = métal alcalin; *X* = P/As). Une nouvelle phase de formulation Na_4_Li_0.62_Co_5.67_Al_0.71_(AsO_4_)_6_ a été synthétisée par réaction à l'état solide.

L'unité asymétrique Na_3_Co*M*1*M*2As_2_O_19_ renferme en plus des deux octaèdres *M*1O_6_ (*M*1=Co_0.29_Al_0.71_) et Co3O_6_, trois tétraèdres As1O_4_, As2O_4_ et *M*2O_4_ (*M*2=Co_0.69_Li_0.31_) où l'un des deux atomes d'arsenic est situé sur le miroir (Fig. 1). Dans la charpente anionique, les dimères Co_2_O_10_ arrangés dans le plan *ac* sont liés entre eux par les tétraèdres As2O_4_, d'une part par partage d'arêtes avec les octaèdres Co3O_6_ et d'autre part par formation de ponts triples (Fig. 2). Au sein de ces chaînes, les fenêtres quadrilatères résultantes sont occupées par les tétraèdres *M*2O_4_ formant ainsi des rubans disposés selon la direction [100] (Fig. 3). La jonction entre ces derniers est assurée par mise en commun de sommets avec les tétraèdres As1O_4_. Elle est aussi renforcée par formation de ponts mixtes *M*1–O–Co3. De plus les polyèdres As1O_4_ et *M*1O_4_, situés entre les rubans sont connectés par partage de sommets. Il en résulte des couches polyédriques disposées parallèlement au plan *ab* (Fig. 4). Ces dernières sont liées entre elles par des ponts mixtes Co3–O–As2 pour former une charpente tridimensionnelle possédant des tunnels, à section hexagonale, disposés selon la direction [100] où logent les cations Na^+^ (Fig. 5).

L'examen des facteurs géométriques dans la structure montre qu'ils sont en bon accord avec ceux rencontrés dans la littérature (Marzouki *et al.*, 2012; Ben Smida *et al.*, 2013; Guesmi & Driss, 2012). D'autre part, le calcul des valences de liaisons (BVS), utilisant la formule empirique de Brown (Brown & Altermatt, 1985), conduit aux valeurs des charges des cations suivants: Na1(0.942), Na2(0.826), Na3(0.942), As1(5.010), As2(4.867), *M*1=Co_0.29_Al_0.71_(2.686), *M*2=Co_0.69_Li_0.31_(1.293) et Co3(1.981).

Un examen rigoureux de différentes structures trouvées dans la littérature révèle que le matériau étudié est un nouveau membre d'une famille de phases incluant Na_4_Ni_7_(PO_4_)_6_ (Moring & Kostiner, 1986), Na_4_Co_7_(PO_4_)_6_ (Kobashi *et al.*, 1998) et K_4_Ni_7_(AsO_4_)_6_ (Ben Smail *et al.*, 1999). Ce dernier cristallise dans le groupe d'espace *C*2/*m* tandis que les phosphates sont revendiqués pour être non-centrosymétriques (groupe d'espace: *Cm*). Mais dans le cas du Xenophyllite Na_4_Fe^II^_7_(AsO_4_)_6_ (Burke *et al.*, 2006) ayant une formulation analogue (type *A*^I^_4_*M*^II^_7_(*X*O_4_)_6_ avec *X* = P/As), il cristallise dans le système triclinique groupe d'espace *P*1.

Une comparaison de la structure de Na_4_Co_4_(Co_0.69_Li_0.31_)_2_(Co_0.29_Al_0.71_)(AsO_4_)_6_ avec celles de l'hagendorfite (Na_0.84_Ca_0.32_)Mn(Fe_1.74_Al_0.26_)(PO_4_)_3_ de type alluaudite (Redhammer *et al.*, 2005), le rosemaryite (Mn_0.366_Na_0.325_)(Fe_0.911_Na_0.088_)(Al_0.708_Fe_0.296_)(Fe_0.791_Al_0.215_)(PO_4_)_3_ (Hatert *et al.*, 2005) et le wyllieite Na_4.6_(CaMn)Fe_4_(Fe_0.75_Mg_0.25_)_4_(Fe_0.25_Al_0.75_)_4_(PO_4_)_12_ (Moore & Molin-Case, 1974) montre que ces derniers cristallisent dans deux groupes d'espace différents (*P*2_1_/*n* (ou *P*2_1_/*c*) et *C*2/*c*) (Fig. 6). Une différence nette dans la charpente a été observée, surtout dans les types de connexion des dimères *M*3_2_O_10_ mis en jeu. En effet, on remarque que dans le cas de l'alluaudite, du rosemaryite et du wyllieite les dimères (Fe/Al)_2_O_10_ sont liés entre eux, seulement par les tétraèdres P2O_4_ par partage de sommets. Dans ce cas, les polyèdres métalliques sont alors à l'extrémité des fenêtres quadrilatères résultantes. D'autre part, pour le wyllieite de formulation développée Na_4.6_(CaMn)Fe_4_(Fe_0.75_Mg_0.25_)_4_(Fe_0.25_Al_0.75_)_4_(PO_4_)_12_, les polyèdres (CaMn)O_6_ renforcent la jonction des octaèdres (Fe/Mg)O_6_ situés en dehors des fenêtres avec les autres dimères *M*3_2_O_10_ (*M*3=Fe/Al).

### 2. Experimental

Un mélange de Na_2_CO_3_ (PROLABO, 27778), LiOH.H_2_O (FLUKA, 62530), Co(NO_3_).6H_2_O (FLUKA, 60832), Al_2_O_3_ (FLUKA, 06285), NH_4_H_2_AsO_4_ (préparé au laboratoire, ASTM 01–775) pris dans les proportions molaires 0.5:1:3:1:3 est mis dans un bécher contenant 15 ml d'eau distillée. Quelques gouttes d'acide nitrique ont été ajoutées pour dissocier totalement les réactifs. La solution est mise à l'étuve à une température de 340 K. Un précipité sous forme de poudre fine et de couleur rose a été obtenu. Ce dernier est finement broyé et mis dans un creuset en silice. Il est préchauffé à 673 K pendant 24 heures afin d'éliminer les produits volatils. Après refroidissement et broyage, le résidu est porté à une température de 1143 K pendant 5 jours. Un refroidissement lent (5°/24 h) a été appliqué jusqu'à 843 K, suivis d'un autre plus rapide (5°/12 h) jusqu'à l'ambiante. Des cristaux de couleur violette sous forme de parallélépipèdes sont apparus sur les parois du creuset. Des monocristaux de taille convenable sont séparés à l'eau bouillante. L'analyse par EDX sur microscope électronique à balayage (type quanta 200, marque FEI) confirme la présence des éléments chimiques attendus notamment Na, Co, Al, As, et l'oxygène. On note que le Li (*Z* < 6) est indétectable par ce type d'analyse.

### 3. Refinement

Au début, les taux d 'occupation des sites *M*1 et *M*2 ont été librement affinés. Le résultat trouvé nous a conduit à les contraindre à 1.00 par l'utilisation d'une contrainte douce (restraint SUMP) avec une valeur sigma petite. L'utilisation de la commande EADP autorisée par le programme *SHELX*, pour les couples d'ions Co1/Al1 et Co2/Li2 conduit à des ellipsoïdes bien définis. De plus, les densités d'électrons maximum et minimum restantes dans la carte de Fourier-différence sont acceptables et sont situées respectivements à 0.76 Å de O4 et à 0.84 Å de As1.

### Crystal data

**Table:**

Na_4_Li_0.62_Co_5.67_Al_0.71_(AsO_4_)_6_	*F*(000) = 1196
*M**_r_* = 1283.06	*D*_x_ = 4.124 Mg m^−^^3^
Monoclinic, *C*2/*m*	Mo *K*α radiation, λ = 0.71073 Å
Hall symbol: -C 2y	Cell parameters from 25 reflections
*a* = 10.7444 (9) Å	θ = 11–15°
*b* = 14.847 (2) Å	µ = 14.22 mm^−^^1^
*c* = 6.7223 (8) Å	*T* = 298 K
β = 105.51 (2)°	Prism, purple
*V* = 1033.3 (2) Å^3^	0.26 × 0.24 × 0.22 mm
*Z* = 2	

### Data collection

**Table:**

Enraf–Nonius CAD-4 diffractometer	1048 reflections with *I* > 2σ(*I*)
Radiation source: fine-focus sealed tube	*R*_int_ = 0.027
Graphite monochromator	θ_max_ = 27.0°, θ_min_ = 2.4°
ω/2θ scans	*h* = −13→13
Absorption correction: ψ scan (North *et al.*, 1968)	*k* = −1→18
*T*_min_ = 0.033, *T*_max_ = 0.042	*l* = −8→2
1615 measured reflections	2 standard reflections every 120 min
1174 independent reflections	intensity decay: 1.4%

### Refinement

**Table:**

Refinement on *F*^2^	Primary atom site location: structure-invariant direct methods
Least-squares matrix: full	Secondary atom site location: difference Fourier map
*R*[*F*^2^ > 2σ(*F*^2^)] = 0.026	*w* = 1/[σ^2^(*F*_o_^2^) + (0.0261*P*)^2^ + 8.122*P*] where *P* = (*F*_o_^2^ + 2*F*_c_^2^)/3
*wR*(*F*^2^) = 0.068	(Δ/σ)_max_ = 0.001
*S* = 1.13	Δρ_max_ = 0.87 e Å^−^^3^
1174 reflections	Δρ_min_ = −0.87 e Å^−^^3^
117 parameters	Extinction correction: *SHELXL*, Fc^*^=kFc[1+0.001xFc^2^λ^3^/sin(2θ)]^-1/4^
2 restraints	Extinction coefficient: 0.00110 (14)

### Special details

**Table:**

Geometry. All e.s.d.'s (except the e.s.d. in the dihedral angle between two l.s. planes) are estimated using the full covariance matrix. The cell e.s.d.'s are taken into account individually in the estimation of e.s.d.'s in distances, angles and torsion angles; correlations between e.s.d.'s in cell parameters are only used when they are defined by crystal symmetry. An approximate (isotropic) treatment of cell e.s.d.'s is used for estimating e.s.d.'s involving l.s. planes.
Refinement. Refinement of *F*^2^ against ALL reflections. The weighted *R*-factor *wR* and goodness of fit *S* are based on *F*^2^, conventional *R*-factors *R* are based on *F*, with *F* set to zero for negative *F*^2^. The threshold expression of *F*^2^ > σ(*F*^2^) is used only for calculating *R*-factors(gt) *etc*. and is not relevant to the choice of reflections for refinement. *R*-factors based on *F*^2^ are statistically about twice as large as those based on *F*, and *R*- factors based on ALL data will be even larger.

### Fractional atomic coordinates and isotropic or equivalent isotropic displacement parameters (Å^2^)

**Table:**

	*x*	*y*	*z*	*U*_iso_*/*U*_eq_	Occ. (<1)
As2	0.59838 (4)	0.82118 (3)	0.79004 (7)	0.00867 (15)	
As1	0.81961 (6)	0.0000	0.56316 (10)	0.01067 (18)	
Co3	0.67950 (6)	0.81829 (4)	0.31816 (9)	0.00996 (17)	
Co2	0.0000	0.83579 (9)	0.5000	0.0144 (5)	0.690 (5)
Li2	0.0000	0.83579 (9)	0.5000	0.0144 (5)	0.310 (5)
Co1	0.5000	0.0000	0.5000	0.0076 (6)	0.286 (10)
Al1	0.5000	0.0000	0.5000	0.0076 (6)	0.714 (11)
Na1	0.9266 (5)	0.8844 (3)	0.0071 (7)	0.0323 (11)	0.50
Na2	0.1821 (6)	0.0000	0.9226 (9)	0.0199 (12)	0.50
Na3	0.4291 (12)	0.0000	−0.0240 (13)	0.066 (4)	0.50
O1	0.4915 (3)	0.7349 (2)	0.7760 (5)	0.0176 (7)	
O2	0.6883 (3)	0.7907 (2)	0.6291 (5)	0.0150 (7)	
O3	0.4874 (3)	−0.0941 (2)	0.7033 (5)	0.0127 (6)	
O4	0.9410 (5)	0.0000	0.7772 (8)	0.0239 (12)	
O5	0.6804 (5)	0.0000	0.6305 (8)	0.0195 (11)	
O6	0.6918 (3)	0.8497 (3)	0.0228 (5)	0.0180 (7)	
O7	0.8273 (4)	−0.0903 (3)	0.4118 (6)	0.0252 (9)	

### Atomic displacement parameters (Å^2^)

**Table:**

	*U*^11^	*U*^22^	*U*^33^	*U*^12^	*U*^13^	*U*^23^
As2	0.0063 (2)	0.0112 (2)	0.0081 (2)	0.00085 (16)	0.00117 (17)	−0.00121 (16)
As1	0.0072 (3)	0.0091 (3)	0.0148 (3)	0.000	0.0014 (2)	0.000
Co3	0.0079 (3)	0.0123 (3)	0.0094 (3)	0.0006 (2)	0.0019 (2)	0.0006 (2)
Co2	0.0101 (7)	0.0177 (8)	0.0154 (7)	0.000	0.0034 (5)	0.000
Li2	0.0101 (7)	0.0177 (8)	0.0154 (7)	0.000	0.0034 (5)	0.000
Co1	0.0055 (10)	0.0075 (10)	0.0097 (10)	0.000	0.0019 (7)	0.000
Al1	0.0055 (10)	0.0075 (10)	0.0097 (10)	0.000	0.0019 (7)	0.000
Na1	0.040 (3)	0.025 (2)	0.025 (2)	−0.009 (2)	−0.003 (2)	0.0085 (19)
Na2	0.023 (3)	0.020 (3)	0.018 (3)	0.000	0.007 (2)	0.000
Na3	0.171 (11)	0.013 (3)	0.025 (4)	0.000	0.044 (7)	0.000
O1	0.0134 (17)	0.0148 (17)	0.0213 (18)	−0.0011 (14)	−0.0010 (14)	0.0044 (14)
O2	0.0170 (17)	0.0176 (17)	0.0129 (16)	0.0088 (14)	0.0085 (13)	0.0025 (13)
O3	0.0100 (14)	0.0109 (15)	0.0175 (16)	0.0019 (12)	0.0043 (12)	−0.0009 (13)
O4	0.015 (2)	0.023 (3)	0.026 (3)	0.000	−0.007 (2)	0.000
O5	0.011 (2)	0.020 (3)	0.028 (3)	0.000	0.006 (2)	0.000
O6	0.0162 (17)	0.0290 (19)	0.0081 (15)	−0.0074 (15)	0.0018 (13)	−0.0028 (14)
O7	0.0240 (18)	0.026 (2)	0.030 (2)	−0.0131 (16)	0.0150 (17)	−0.0160 (17)

### Geometric parameters (Å, º)

**Table:**

As2—O6^i^	1.672 (3)	Co1—O3^iii^	1.984 (3)
As2—O2	1.694 (3)	Co1—O3	1.984 (3)
As2—O1	1.706 (3)	Co1—O3^x^	1.984 (3)
As2—O3^ii^	1.724 (3)	Na1—O1^iv^	2.314 (6)
As1—O4	1.663 (5)	Na1—O4^xi^	2.342 (6)
As1—O5	1.674 (5)	Na1—O4^xii^	2.443 (6)
As1—O7	1.698 (4)	Na1—O1^xiii^	2.573 (6)
As1—O7^iii^	1.698 (4)	Na1—O6	2.605 (6)
Co3—O7^ii^	2.056 (4)	Na2—O4^xiv^	2.512 (8)
Co3—O6	2.078 (3)	Na2—O6^xv^	2.585 (5)
Co3—O2	2.107 (3)	Na2—O6^xvi^	2.585 (5)
Co3—O2^iv^	2.119 (3)	Na2—O7^v^	2.596 (6)
Co3—O1^v^	2.165 (3)	Na2—O7^x^	2.596 (6)
Co3—O3^vi^	2.188 (3)	Na2—O4^xvii^	2.692 (8)
Co2—O7^vi^	2.100 (4)	Na2—O5^xvii^	2.972 (8)
Co2—O7^vii^	2.100 (4)	Na3—O3^xviii^	2.513 (8)
Co2—O1^viii^	2.155 (4)	Na3—O3^xix^	2.513 (8)
Co2—O1^ix^	2.155 (4)	Na3—O3^x^	2.524 (8)
Co1—O5	1.902 (5)	Na3—O3^v^	2.524 (8)
Co1—O5^x^	1.902 (5)	Na3—O6^xx^	2.583 (7)
Co1—O3^v^	1.984 (3)	Na3—O6^xxi^	2.583 (7)
			
O6^i^—As2—O2	111.31 (17)	O2—Co3—O3^vi^	89.99 (13)
O6^i^—As2—O1	117.96 (17)	O2^iv^—Co3—O3^vi^	164.89 (13)
O2—As2—O1	104.74 (18)	O1^v^—Co3—O3^vi^	72.81 (12)
O6^i^—As2—O3^ii^	108.63 (17)	O7^vi^—Co2—O7^vii^	117.0 (2)
O2—As2—O3^ii^	116.12 (16)	O7^vi^—Co2—O1^viii^	105.07 (14)
O1—As2—O3^ii^	97.73 (16)	O7^vii^—Co2—O1^viii^	104.44 (13)
O4—As1—O5	108.4 (3)	O7^vi^—Co2—O1^ix^	104.44 (13)
O4—As1—O7	111.45 (18)	O7^vii^—Co2—O1^ix^	105.07 (14)
O5—As1—O7	110.63 (16)	O1^viii^—Co2—O1^ix^	121.69 (19)
O4—As1—O7^iii^	111.45 (18)	O5—Co1—O5^x^	180.00 (13)
O5—As1—O7^iii^	110.63 (16)	O5—Co1—O3^v^	93.93 (15)
O7—As1—O7^iii^	104.2 (3)	O5^x^—Co1—O3^v^	86.07 (15)
O7^ii^—Co3—O6	84.43 (15)	O5—Co1—O3^iii^	86.07 (15)
O7^ii^—Co3—O2	89.93 (15)	O5^x^—Co1—O3^iii^	93.93 (15)
O6—Co3—O2	173.76 (14)	O3^v^—Co1—O3^iii^	180.0
O7^ii^—Co3—O2^iv^	91.41 (15)	O5—Co1—O3	86.07 (15)
O6—Co3—O2^iv^	96.91 (14)	O5^x^—Co1—O3	93.93 (15)
O2—Co3—O2^iv^	80.51 (14)	O3^v^—Co1—O3	90.49 (19)
O7^ii^—Co3—O1^v^	173.12 (14)	O3^iii^—Co1—O3	89.51 (19)
O6—Co3—O1^v^	96.60 (14)	O5—Co1—O3^x^	93.93 (15)
O2—Co3—O1^v^	89.32 (14)	O5^x^—Co1—O3^x^	86.07 (15)
O2^iv^—Co3—O1^v^	95.21 (13)	O3^v^—Co1—O3^x^	89.51 (19)
O7^ii^—Co3—O3^vi^	100.36 (14)	O3^iii^—Co1—O3^x^	90.49 (19)
O6—Co3—O3^vi^	93.66 (14)	O3—Co1—O3^x^	180.0

Symmetry codes: (i) *x*, *y*, *z*+1; (ii) *x*, *y*+1, *z*; (iii) *x*, −*y*, *z*; (iv) −*x*+3/2, −*y*+3/2, −*z*+1; (v) −*x*+1, *y*, −*z*+1; (vi) −*x*+1, *y*+1, −*z*+1; (vii) *x*−1, *y*+1, *z*; (viii) −*x*+1/2, −*y*+3/2, −*z*+1; (ix) *x*−1/2, −*y*+3/2, *z*; (x) −*x*+1, −*y*, −*z*+1; (xi) *x*, *y*+1, *z*−1; (xii) −*x*+2, −*y*+1, −*z*+1; (xiii) *x*+1/2, −*y*+3/2, *z*−1; (xiv) *x*−1, *y*, *z*; (xv) −*x*+1, −*y*+1, −*z*+1; (xvi) −*x*+1, *y*−1, −*z*+1; (xvii) −*x*+1, −*y*, −*z*+2; (xviii) *x*, *y*, *z*−1; (xix) *x*, −*y*, *z*−1; (xx) −*x*+1, *y*−1, −*z*; (xxi) −*x*+1, −*y*+1, −*z*.

## References

[bb1] Alvarez-Vega, M., Gallardo-Amores, J. M., Garcia-Alvarado, F. & Amador, U. (2006). *Solid State Sci.* **8**, 952–957.

[bb2] Aurivillius, B., Lindblom, C. I. & Stenson, P. (1964). *Acta Chem. Scand.* **18**, 1555–1557.

[bb3] Ben Smail, R., Driss, A. & Jouini, T. (1999). *Acta Cryst.* C**55**, 284–286.

[bb4] Ben Smida, Y., Guesmi, A. & Driss, A. (2013). *Acta Cryst.* E**69**, i39. 10.1107/S1600536813013548 PMC3684868 23794970

[bb5] Brandenburg, K. (1998). *DIAMOND.* University of Bonn, Germany.

[bb6] Brown, I. D. & Altermatt, D. (1985). *Acta Cryst.* B**41**, 244–247.

[bb7] Burke, E. A. J., Ferraris, G. & Hatert, F. (2006). *New minerals approved in 2006.* Nomenclature modifications approved in 2006 by the Commission on New Minerals, Nomenclature and Classification, International Mineralogical Association. http://pubsites.uws.edu.au/ima-cnmnc/minerals2006.pdf

[bb8] Duisenberg, A. J. M. (1992). *J. Appl. Cryst.* **25**, 92–96.

[bb9] Farrugia, L. J. (2012). *J. Appl. Cryst.* **45**, 849–854.

[bb10] Frigui, W., Zid, M. F. & Driss, A. (2012). *Acta Cryst.* E**68**, i40–i41. 10.1107/S1600536812018843 PMC3379051 22719272

[bb11] Goodenough, J. B., Borel, H., Hong, Y.-P. & Kafalas, T. A. (1976). *Mater. Res. Bull.* **11**, 203–220.

[bb12] Guesmi, A. & Driss, A. (2012). *Acta Cryst.* E**68**, i58. 10.1107/S1600536812027791 PMC3393142 22807699

[bb13] Harms, K. & Wocadlo, S. (1995). *XCAD4* University of Marburg, Germany.

[bb14] Hatert, F., Lefevre, P., Fransolet, A. M., Spirlet, M.-R., Rebbouh, L., Fontan, F. & Keller, P. (2005). *Eur. J. Mineral.* **17**, 749–759.

[bb15] Keller, P., Riffel, H., Zettler, F. & Hess, H. (1981). *Z. Anorg. Allg. Chem.* **474**, 123–134.

[bb16] Kobashi, D., Kohara, S., Yamakawa, J. & Kawahara, A. (1998). *Acta Cryst.* C**54**, 7–9.

[bb17] Macíček, J. & Yordanov, A. (1992). *J. Appl. Cryst.* **25**, 73–80.

[bb18] Marzouki, R., Guesmi, A., Zid, M. F. & Driss, A. (2012). *Cryst. Struct. Theor. Appl.* **1**, 68-73.

[bb19] Moore, P. B. & Molin-Case, J. (1974). *Am. Mineral.* **59**, 280–290.

[bb20] Moring, J. & Kostiner, E. (1986). *J. Solid State Chem.* **62**, 105–111.

[bb21] Nagpure, M., Shinde, K. N., Kumar, V., Ntwaeaborwa, O. M., Dhoble, S. J. & Swart, H. C. (2010). *J. Alloys Compd*, **492**, 384–388.

[bb22] North, A. C. T., Phillips, D. C. & Mathews, F. S. (1968). *Acta Cryst.* A**24**, 351–359.

[bb23] Prabaharan, S. R. S., Michael, M. S., Radhakrishna, S. & Julien, C. (1997). *J. Mater. Chem.* **7**, 1791–1796.

[bb24] Redhammer, G. J., Tippelt, G., Bernroider, M., Lottermoser, W., Amthauer, G. & Roth, G. (2005). *Eur. J. Mineral.* **17**, 915–932.

[bb25] Sheldrick, G. M. (2008). *Acta Cryst.* A**64**, 112–122. 10.1107/S0108767307043930 18156677

